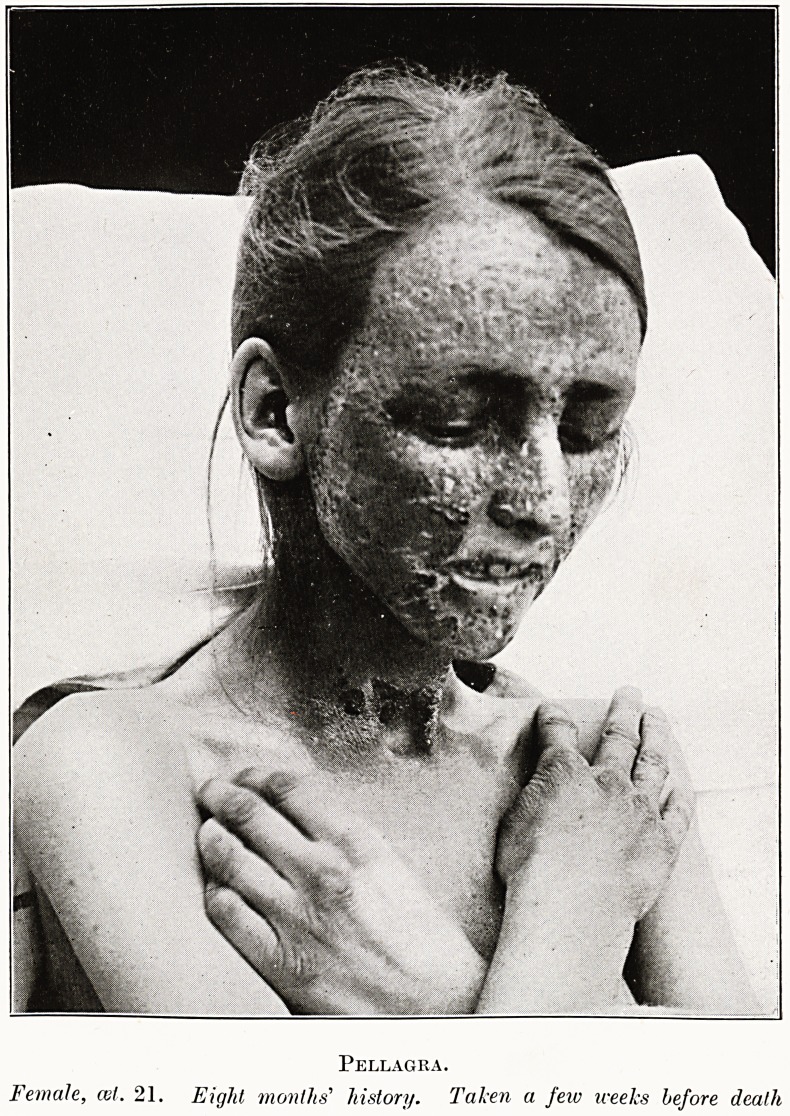# Observations on Pellagra

**Published:** 1927

**Authors:** E. Barton White, Geoffrey Hadfield

**Affiliations:** Medical Superintendent, Bristol Mental Hospital; Pathologist to the General Hospital, Bristol


PLATE III.

PELLAGRA.

*Female, æt. 21. Eight months' history. Taken a few weeks before death*

### OBSERVATIONS ON PELLAGRA.

### WITH AN ACCOUNT OF A CASE.

BY

E. Barton White, M.R.C.S. (Eng.), L.R.C.P. (Lond.),
*Medical Superintendent, Bristol Mental Hospital,*

AND

Geoffrey Hadfield, M.D., M.R.C.P. (Lond.),
*Pathologist to the General Hospital, Bristol.*

---

### Introduction.

Pellagra is a disease of considerable interest to those having the care of mental patients, as in the British Isles it is almost confined to those mental patients who refuse food. It is not a result of mere starvation, for it is rare amongst cases of obstructive gastro-intestinal cancer, however chronic. Nevertheless, its origin appears to be dietetic and its primary lesion gastro-intestinal. With the majority of observers we consider the primary fault to be a state of avitaminosis and the visceral manifestations to be the result of a toxæmia predisposed to by the dietetic deficiency. The disease has a valuable external sign—a typical dermatitis due to an abnormal sensitiveness to ultra-violet light. It is curable in the early stages by suitable dieting, but when once established neither this nor any other treatment seems to avert a fatal issue. In the care of the insane, therefore, it is of considerable importance, first, to look on all patients who consistently refuse food as potential pellagrins, whilst realising that only a small percentage are susceptible; second, to regard gastro-intestinal symptoms, especially soreness of the tongue and attacks of diarrhœa, in such patients as laying them under suspicion; and, third, to regard an excessive reaction to ultra-violet light as a sufficient indication for vigorous dietetic measures. Two cases of the disease have recently occurred at the Bristol Mental Hospital, Fishponds, one of which we report in this article.

### CASE REPORT.

**Abstract of history and clinical notes.**

Young married woman in fairly comfortable circumstances, aged 21, married about two years, one child nearly 12 months old, breast fed. No family history of insanity or alcoholism: other members of family healthy and normal. Has not been out of the South-West of England, and has enjoyed good health. She was nervous and depressed after her child was born, and for the last few weeks would sit staring in front of her, lost interest in her home and neglected it. She began to feel that she was not able to nurse the child, and acquired the delusion that she was slowly killing it, and that death was to be her punishment. She was sleeping badly and took very little food. Admitted in February, 1926.

She was a blonde with a clear pink and white complexion and blue eyes. The skin was rather dry and waxy. She was in a reduced condition, with poor muscular tone and signs of recent wasting. Height 5 ft. 1 in., and weight 6 st. 5 lb. Head well shaped. No stigmata except the hands, which were long and narrow with little thenar and hypothenar eminences and the thumb only slightly opposed to the fingers—suggesting the simian hand. The teeth were well shaped, but several were carious and the gums, and mouth generally, in a dirty condition, chiefly from neglect. The tongue was dry and red. The lips were dry. The stomach was not dilated, and examination of the abdomen showed nothing abnormal. Menstruation was said to be scanty and irregular. Urine, sp. gr. 1022, no albumen or sugar. Heart and lungs were normal. The pulse was 80 and temperature 97·0. The thyroid was normal to palpation, not enlarged, and symmetrical. All tendon reflexes were exaggerated; plantar response flexor; abdominal reflexes present. The pupils were dilated, regular and equal and very sensitive to light. There was apparent anæsthesia to pin-pricks over the trunk and limbs but not on the dorsum of hands. This could only be tested by muscular response, as she was in a state of stupor. A month later there was fair co-ordination of movements. She showed marked catatonia, the limbs remaining in any placed position. Her mental state was one of catatonic stupor. Her expression was vacant. She would react to no stimulus, but would swallow liquids from a feeding cup. She was incontinent, and could do nothing for herself. She was put to bed on a verandah facing south. After ten days she began to look about her and whispered a few replies to questions. She began to be restless during the night, and would sit up in bed and appear distressed: conversations and suggestion pacified her for a time, and she began to take solid food. She spoke readily, but in monotone. She then displayed ideas of apprehension, and had the delusion that she was to be buried alive or put in a boiling bath to kill her, and begged not to be taken to the bathroom. She asked for reassurance, but would accept none.

It was thought that this was a case of *folie à deux*, as the patient in the next bed on the verandah had been giving expression to the same distressing ideas for some time. This patient was removed, and to some extent these ideas faded after a fortnight. She was now thought to be a case of dementia præcox. She began to repeat her delusions automatically and without emotion, and would cry out loudly at times, though not distressed. Early in May she became sunburned about the face and neck in common with others on the verandah. When dull weather supervened this did not fade as in the others, but became dusky red with inflamed margins slowly spreading. Brownish pigmentation appeared, particularly at the edges. The dorsa of the hands, which were often covered by the bedclothes, were less affected, but the same condition appeared about the joints. The skin of the face became raised and thickened and more deeply pigmented in patches. A few suppurating bullæ appeared and remained as open septic sores. The lips were dry and had septic cracks. In other places the skin became thickened and scaly and more darkly pigmented. The dorsum of the hands showed thickened, pigmented and scaly patches over the knuckles, showing the condition described as dermatogra. There were ulcers inside the lips and cheek. She was resistive and difficult to feed. Towards the end of May she had periodic attacks of diarrhœa with liporrhœa and creatorrhœa, which would last two or three days. She became very emaciated and exhausted. The temperature rose to 103°, and she vomited on several occasions. She showed signs of septic absorption, and gradually sank and died on June 15th, 1926. Cultures from the stool showed no pathogenic organisms. Sugar tolerance tests were normal, and the blood urea was not increased.

### Abstract of post-mortem notes.

*Externally.*—Considerable emaciation. Skin rash present as described in clinical notes, with considerable recent acute dermatitis superimposed.

*Tongue.*—Smooth and atrophic with several shallow linear ulcers.

*Thyroid and Parathyroid* showed no abnormality in size, weight or appearance, and were normal histologically. Some general enlargement of cervical lymphatic glands, but no softening or suppuration.

*Thorax.*—Thymus gland atrophic and practically all fat. No abnormality in the heart beyond terminal softening and friability of myocardium. Bases of both lungs showed hypostatic congestion, but were otherwise normal.

*Abdomen.*— *Stomach* normal in size and shape. Moderate degree of atrophic gastritis, rugæ ill-defined, inner surface smooth, and walls definitely thinner than normal.

*Small bowel.*—Externally, walls thin and fragile. Internally, valvulæ conniventes thin, and mucous membrane pale, sodden and dusted with small hemorrhagic points : no ulceration, no peritonitis, no enlargement of mesenteric glands.

*Large bowel* shows same condition of general atrophy, and "tissue-paper" walls, except for last foot of colon, where the mucous membrane is denuded and there is shallow superficial ulceration with a thin greyish diffuse slough and a few points and streaks of hemorrhage. Strips of the bowel wall four inches long and an inch wide were cut from various regions, and after being rolled up were tied, embedded in paraffin wax and cut. These were compared with similar sections taken from a normal control. As is usual in post-mortem material, the mucous membrane in both series showed considerable surface desquamation of its cells, but it could be easily established that this was more marked in the pellagra specimen. With this change there was a generalised but moderate atrophy of the submucous lymphoid tissue ; the follicles were small and ill-defined, and the cells less closely packed than normal. There was definite congestion in the submucous coat, and in this layer and in the supporting tissue of the villi there was a constant and striking diminution in the number of leucocytes and wandering cells. Although these observations were controlled, we are aware that there are fallacies in accepting such appearances as these as part of the morbid anatomy of the disease, as sections of the small bowel vary so much with the state of its activity ; but we feel satisfied that this generalised atrophy, especially of the lymphoid elements, and the lack of phagocytic cells in the sub-mucosa are more than can be accounted for by assuming that the mucous membrane was in the normal " resting " condition. The changes in the colon were of recent date and acute in type.

*Spleen.*—Not increased in size. Pulp rather soft but not suggestive of acute infection.

*Liver, bile passages, pancreas.*—No abnormalities were detected in these organs. Histologically no abnormality was found in the liver except the changes associated with recent acute toxic degeneration of its cells, in all probability produced during the terminal phases of the disease.

*The suprarenal glands* showed no abnormality in size or weight and appeared normal histologically.

No abnormalities were discovered in the genito-urinary organs.

*Nervous System.*—The peripheral nervous system was not examined. Sections of the spinal cord and cortex were stained for degenerated myelin by the Weigert-Pal and Marchi methods and for neuroglia and nerve cells. The changes found were slight. No conclusive nerve cell degeneration could be demonstrated, but in several sections from the mid-dorsal cord small areas of degeneration were constantly seen deep in the posterior columns in the tract of Goll, and always in the same position. When examined by the Marchi method the foci were found to be composed of scattered irregular areas of degenerating myelin, between which were many intact sheaths. The insignificance of the changes found, although consistent with the clinical condition, struck us very forcibly, as previous experience of these cases has always resulted in the discovery of much grosser changes. No neurogliosis was found.

*Pituitary body* seemed to be rather larger than normal on naked-eye examination, but histologically there was no reason for supposing that the glandular activity was in any way in excess of normal.

### Remarks on Reported Case.

As is usual in this disease, the light-dermatitis first suggested the diagnosis. Later this became typical in appearance and character and, being accompanied by the typical stomatitis, a red, glazed tongue and attacks of diarrhœa, warranted a confident diagnosis. The only other skin condition resembling pellagra is the type described by McCall Anderson as associated with porphyrinuria. In this the destruction of skin is greater and the urine deeply pigmented. At no time was any abnormality in the colour or chemistry of the urine found. When it was observed that the dermatitis progressed even when the skin was covered and the patient kept indoors, a thorough examination of the nervous system was undertaken, especially with the object of detecting minor degrees of loss of conduction in the posterior columns of the cord. No physical signs of this could be detected at any time, but the difficulties in the examination of the sensory system in the insane are notorious.

Compared with the average case, the course was rapid and the emaciation and diarrhœa severe. It is well known that almost at any time a pellagrin may lapse into a typhoid-like state and die in a few weeks, and we assume that in this case the terminal phase of the disease supervened before the changes in the spinal cord had become established.

It seems very probable that long before the light-dermatitis develops changes in the gastro-intestinal tract are taking place, and as a rule some degree of stomatitis or diarrhœa antedates the skin lesion, whilst both dermatitis and diarrhœa almost always antedate nervous signs and symptoms. As this patient was admitted in the winter, it is likely that there may have been a period of several months during which minor gastro-intestinal symptoms were present.

As regards the dementia, it seems extremely likely that this was not the mental state proper to pellagra, which is found during the latter half of the disease in patients with the dermatitis and gastro-intestinal symptoms fully developed; but, as is usual in this country, during a period of mental ill-health characterised by the refusal to take food, the changes in the bowel characteristic of the first stages of the disease were established. The course of the disease and the relatively meagre post-mortem findings leave us in no doubt that the case was one of pellagra of a rather unusually acute type.

### DISCUSSION.

*The preliminary avitaminosis.*—Most, though not all, authorities favour the view that a period of avitaminosis precedes the onset of the disease. The following considerations support this view:—

(*a*) Pellagra is always preceded either by poverty or by refusal or inability to take food. When it is preceded by poverty the provocative diet is usually composed of one article of food only (*e.g.* maize or rice).

(*b*) It is a general principle in the case of experimental animals that the administration of the defective vitamin cures avitaminosis in the early stages but is ineffective later. This agrees with clinical experience in pellagra.

(*c*) Gastro-intestinal atrophy has been shown by McCarrison and by Cramer to be a frequent accompaniment of states of experimental avitaminosis. This agrees with the post-mortem findings in pellagra.

*The vitamin concerned.*—We may rule out the large group of vitamins concerned with growth in young animals, *i.e.* most of the fat-soluble group (A and B), and probably the water-soluble, anti-scorbutic vitamin. The remaining group, water-soluble B, contains two vitamins essential to normal nutrition at all ages, one a growth-promoting vitamin, the other anti-neuritic. It is significant that neither of these vitamins can be stored in large amounts in adult tissue as the fat soluble factors can, and that symptoms follow fairly soon on their withdrawal from the diet of experimental animals, whilst continued deprivation is invariably fatal with progressive loss of weight. These vitamins are contained in all natural food-stuffs, chiefly in plant seeds, birds' eggs and yeast. They seem to be always associated with purine and pyrimidine bases, and may be concerned with nuclear metabolism. Deprivation of this group of vitamins is definitely associated with gastro-intestinal atrophy and defective fat absorption, and notoriously with neuritic disease such as avian polyneuritis, which is now recognised as the equivalent of human beri-beri.

*Onset of Pellagra.*—A gastro-intestinal onset is usual. With the initial stomatitis and smooth, sore tongue, achlorhydria has been frequently reported. Attacks of diarrhœa are practically always present. In these respects the disease has several points of contact with pernicious anæmia, and it is not at all unlikely that the oligocythæmia and non-systematised degeneration of the cord found in both conditions is due to failure of the stomach to sterilise its contents from lack of acid, and to toxic absorption from the small bowel. It is interesting to note in this connection that pellagra has been reported to have accompanied six cases of gastric cancer, another condition which, especially when it runs a chronic course, is liable to give rise to sub-acute degeneration of the cord.

*The Spinal Cord Lesion.* — Kinnier Wilson has described a scattered irregular degeneration of the myelin sheaths of the peripheral nerves, degenerative changes in the cells of Clarke's column, and pseudo-systematised degeneration of the spinal cord most marked in the column of Goll and the marginal zones. He found a singular absence of inflammatory changes. Clinically, there is a general tendency to describe the nervous manifestations as occurring late in the disease. In our experience the nervous manifestations may be surprisingly slight in the more acute cases. The spinal cord lesion of lathyrism, which occurs in India during famine years, resembles that of pellagra, which in turn is usually held to be indistinguishable from sub-acute combined degeneration of pernicious anæmia. The mechanism of infection of the spinal cord is suggested by the work of Orr and Rows, who demonstrated in animals a free lymphatic pathway capable of transporting bacterial toxins from the general peritoneal cavity via the perineural lymphatics to the spinal cord. The severity of the lesions in the cord compared with those of the peripheral nerves is in accord with the principle that nerve fibres in the substance of the central nervous system are more vulnerable than those in peripheral nerves.

*The Skin Lesion.*—Undue sensitiveness to ultra-violet light is typical of pellagra. In the case reported it resulted in a rapidly spreading severe dermatitis. In the more chronic cases it improves very considerably during the winter. This symptom recalls the light-sensitiveness found in congenital porphyrinuria, but absent in the acquired hæmato-porphyrinuria of sulphonal poisoning. Porphyrin is a structural unit common to all hæmoglobins and chlorophylls; it is iron-free and composed of four substituted pyrrhol roups. One variety, coproporphyrin, is normally present in the bowel, and it may be that the atrophied gastro-intestinal wall in pellagra becomes pervious to this pigment and excessive quantities are absorbed. We feel strongly that in this country at least, and in asylum practice in particular, too much stress should not be laid on the presence of the skin lesion in its typical form in making a provisional diagnosis of pellagra. The typical tongue and mouth lesions, combined with bouts of diarrhœa, in an emaciated patient refusing food are sufficient for a provisional diagnosis. It is needless to add how easy it may be to miss these signs in an insane patient. The treatment should include a review of the vitamin content of the feeds, with the addition of egg-yolk and yeast and the administration of hydrochloric acid.

## Figures and Tables

**Figure f1:**